# Comparative 6-Month Wild-Type and Delta-Variant Antibody Levels and Surrogate Neutralization for Adults Vaccinated with BNT162b2 versus mRNA-1273

**DOI:** 10.1128/spectrum.02702-21

**Published:** 2022-03-07

**Authors:** Brian Grunau, Liam Golding, Martin A. Prusinkiewicz, Michael Asamoah-Boaheng, Richard Armour, Ana Citlali Marquez, Agatha N. Jassem, Vilte Barakauskas, Sheila F. O’Brien, Steven J. Drews, Scott Haig, Pascal M. Lavoie, David M. Goldfarb

**Affiliations:** a Centre for Health Evaluation & Outcome Sciences, University of British Columbia, Vancouver, British Columbia, Canada; b Department of Emergency Medicine, University of British Columbia, Vancouver, British Columbia, Canada; c British Columbia Emergency Health Services, Vancouver, British Columbia, Canada; d Department of Obstetrics and Gynecology, University of British Columbia, Vancouver, British Columbia, Canada; e Department of Pediatrics, University of British Columbia, Vancouver, British Columbia, Canada; f Faculty of Medicine, Clinical Epidemiology, Memorial University of Newfoundland, St. John’s, Newfoundland, Canada; g Department of Pathology and Laboratory Medicine, University of British Columbia, Vancouver, British Columbia, Canada; h Public Health Laboratory, British Columbia Centre for Disease Control, Vancouver, British Columbia, Canada; i Canadian Blood Services, Ottawa, Ontario, Canada; j Laboratory Medicine and Pathology, University of Alberta, Edmonton, Alberta, Canada; Pontificia Universidad Católica de Chile; Rega Institute

**Keywords:** SARS-CoV-2, Delta, spike, COVID-19

## Abstract

While mRNA vaccines are highly efficacious against short-term COVID-19, long-term immunogenicity is less clear. We compared humoral immunogenicity between BNT162b2 and mRNA-1273 vaccines 6 months after the first vaccine dose, examining the wild-type strain and multiple Delta-variant lineages. Using samples from a prospective observational cohort study of adult paramedics, we included COVID-19-negative participants who received two BNT162b2 or mRNA-1273 vaccines, and provided a blood sample 170 to 190 days post first vaccine dose. We compared wild-type spike IgG concentrations using the Mann-Whitney U test. We also compared secondary outcomes of: receptor binding domain (RBD) wild-type antibody concentrations, and inhibition of angiotensin-converting enzyme 2 (ACE-2) binding to spike proteins from the wild-type strain and five Delta-variant lineages. We included 571 adults: 475 BNT162b2 (83%) and 96 mRNA-1273 (17%) vaccinees, with a mean age of 39 (SD = 10) and 43 (SD = 10) years, respectively. Spike IgG antibody concentrations were significantly higher (*P* < 0.0001) for those who received mRNA-1273 (GM 601 BAU/mL [GSD 2.05]) versus BNT162b2 (GM 375 BAU/mL [GSD 2.33) vaccines. Results of RBD antibody comparisons (*P* < 0.0001), and inhibition of ACE-2 binding to the wild-type strain and all tested Delta lineages (all *P* < 0.0001), were consistent. Adults who received two doses of mRNA-1273 vaccines demonstrated improved wild-type and Delta variant-specific humoral immunity outcomes at 6 months compared with those who received two doses of the BNT162b2 vaccine.

**IMPORTANCE** The BNT162b2 and mRNA-1273 mRNA SARS-CoV-2 vaccines have demonstrated high efficacy for preventing short-term COVID-19. However, comparative long-term effectiveness is unclear, especially pertaining to the Delta variant. We tested virus-specific antibody responses 6 months after the first vaccine dose and compared individuals who received the BNT162b2 and mRNA-1273 SARS-CoV-2 vaccines. We found that individuals who received the mRNA-1273 vaccine demonstrated superior serological markers at 6 months in comparison with those who received the BNT162b2 vaccine.

## INTRODUCTION

Clinical trials have found the BNT162b2 and mRNA-1273 vaccines to be highly efficacious against short-term severe COVID-19 (1, 2). However, as clinical trial testing was performed against placebo, there is less clarity on the comparative long-term effectiveness of these two mRNA vaccines.

Existing clinical trial testing was performed when the prototypic wild-type strain was predominant (1, 2). The Delta variant (B.1.617.2) was first detected in late 2020, and spread rapidly, becoming the dominant global strain by mid-2021 (3). This variant was found to have an increased risk of hospitalization and death, in comparison to the wild-type strain (4). On December 14, 2021, the Delta variant accounted for 99.2% of COVID-19 cases globally (5). Delta strain mutations resulted in multiple Delta variant lineages with variable predominance throughout different regions of the world (6). While several studies have compared disease severity and immunogenicity of the Delta strain with other strains, differences in vaccine effectiveness among the individual Delta variant lineages is less clear (4, 7–9).

The comparative long-term immunogenicity conveyed from different mRNA vaccines, particularly in relation to the Delta variant is unclear. Further, it is unclear if vaccine-based immunogenicity differences are consistent among the multiple Delta variant lineages; or alternatively, whether differing vaccination strategies may be required depending on which Delta variant is most prevalent in a community. For these reasons, we compared humoral immunogenicity between BNT162b2 and mRNA-1273 vaccinees at 6 months after the first dose, examining the wild-type strain and several different Delta variant lineages.

## RESULTS

### Participant characteristics.

The study included 571 adults (enrolled January 22, 2021 to October 7, 2021); 475 (83%) and 96 (17%) received two doses of the BNT162b2 vaccine or two doses of the mRNA-1273 vaccine, respectively. BNT162b2 and mRNA-1273 vaccinees demonstrated similar mean age and sex distribution (Table 1). The average vaccine dosing interval was 7 weeks in both groups, with similar timing of doses and blood sampling.

**TABLE 1:** Participant characteristics^*a*^

Characteristics	BNT162b2 (*n* = 475)	mRNA-1273 (*n* = 96)
Age (yr), mean (SD)^*b*^	39 (10)	43 (10)
Female sex, n (%)	219 (46)	39 (41)
Vaccination		
January 1, 2021 to 1st vaccine interval (d), mean (SD)	16 (15)	21 (19)
1st vaccine to blood sample interval (d), mean (SD)	182 (4.5)	183 (4.5)
2nd vaccine to blood sample interval (d), mean (SD)	52 (34)	52 (26)
Vaccine dosing interval (d), mean (SD)	51 (37)	52 (26)
Past medical history		
Hypertension, n (%)	31 (6.5)	14 (15)
Diabetes, n (%)	8 (1.7)	2 (2.1)
Asthma, n (%)	62 (13)	15 (16)
Lung disease, n (%)	4 (0.84)	0
Heart disease, n (%)	3 (0.63)	1 (1.0)
Kidney disease, n (%)	1 (0.21)	0
Liver disease, n (%)	7 (1.5)	1 (1.0)
Cancer, n (%)	9 (1.9)	4 (4.2)
Hematologic disease, n (%)	7 (1.5)	0
Immune disease, n (%)	11 (2.3)	2 (2.1)
Neurological disease, n (%)	4 (0.84)	0

a Participants answered the question “Have you been diagnosed by a physician with any of the following chronic medical conditions? (select all that apply).”

b Yr, year; SD, standard deviation; n, number; d, day.

### Primary outcome comparison.

**FIG 1:**
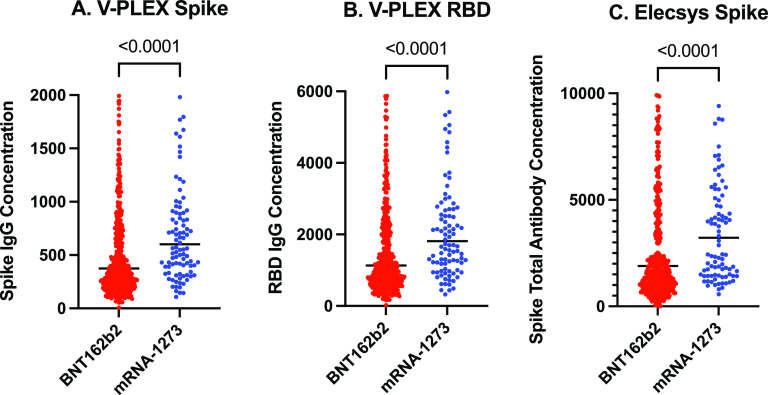
Comparison of spike and receptor-binding domain antibody concentrations (BAU/mL), stratified by mRNA vaccine type. (A) Spike IgG antibody concentrations (BAU/mL), measured on the V-PLEX assay. (B) Receptor-binding domain (RBD) IgG antibody concentrations (BAU/mL), measured on the V-PLEX assay. (C) Spike total antibody concentrations (BAU/mL), measured on the Elecsys assay. The black line represents the geometric mean. *P* values derived from the Mann-Whitney U test. BAU/mL, binding antibody units per mL.

Spike IgG antibody concentrations, measured on the V-PLEX assay (Fig. 1), were significantly higher (*P* < 0.0001) for mRNA-1273 (GM 601 BAU/mL [GSD 2.05]) versus BNT162b2 (GM 375 BAU/mL [GSD 2.33]) vaccinees.

### Secondary outcome comparisons.

Spike total antibody concentrations were significantly higher for mRNA-1273, in comparison with BNT162b2, vaccinees when measured on the Elecsys assay (GM 3223 BAU/mL [GSD 2.36] versus GM 1895 U/mL [GSD 3.12]; *P* < 0.0001). Receptor binding domain (RBD) antibody concentrations were significantly higher for mRNA-1273, in comparison with BNT162b2, vaccinees (GM 1815 BAU/mL [GSD 2.05] versus GM 1131 BAU/mL [GSD 2.33]; *P* < 0.0001).

**FIG 2:**
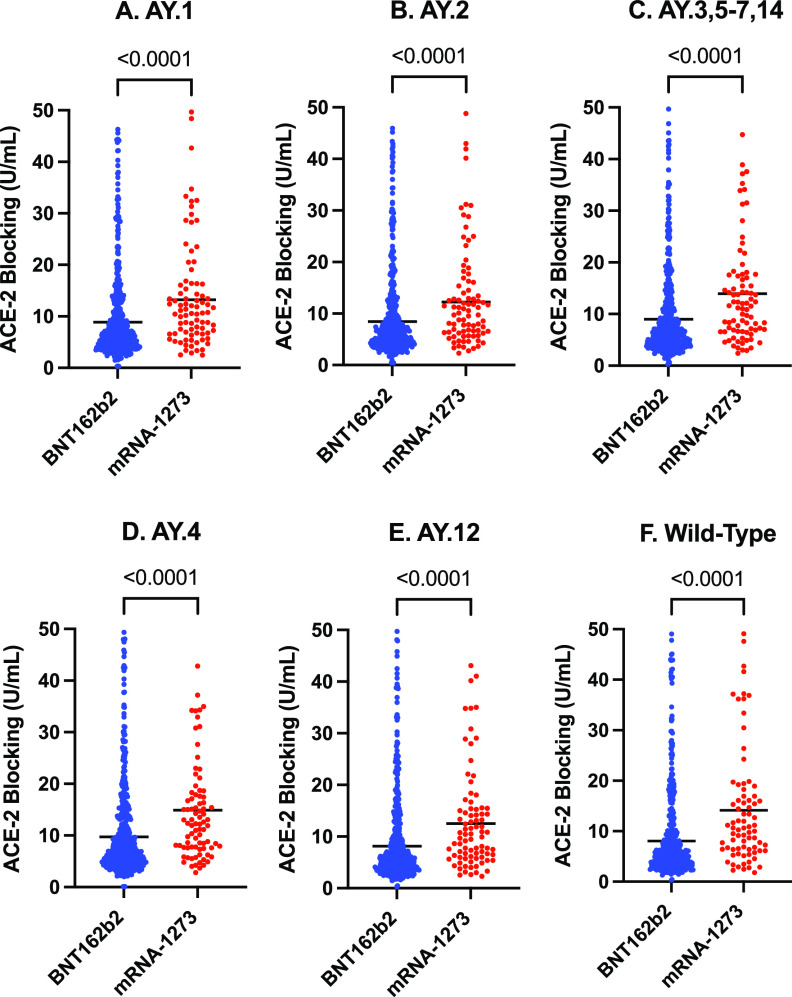
Comparison of inhibition of ACE-2 binding to Delta variant and wild-type strain spike protein concentrations (U/mL), stratified by mRNA vaccine type. Delta variant spike proteins: A. AY.1.; B. AY.2.; C. B.1.617.2/AY.3/AY.5/AY.6/AY.7/AY.14; D. B.1.617.2/AY.4.; E. AY.12. Wild-Type Spike Protein results in Panel F. The black line represents the geometric mean. *P* values derived from the Mann-Whitney U test. U/mL, units per mL.

Inhibition of ACE-2 binding was significantly higher for mRNA-1273, in comparison with BNT162b2 vaccinees, respectively, for the: wild-type strain (GM 14.13 U/mL [GSD 2.74] versus GM 8.06 U/mL [GSD, 3.03]; *P* < 0.0001); as well as for the AY.1 (GM 13.24 U/mL [GSD 2.47] versus GM 8.85 U/mL [GSD 2.76]; *P* < 0.0001), AY.2 (GM 12.21 U/mL [GSD 2.41] versus GM 8.43 U/mL [GSD, 2.67]; *P* < 0.0001), B.1.617.2/AY.3/AY.5/AY.6/AY.7/AY.14 (GM 13.95 U/mL [GSD 2.56] versus GM 8.97 U/mL [GSD, 2.86], *P* < 0.0001), B.1.617.2/AY.4 (GM 14.90 U/mL [GSD 2.39] versus GM 9.71 U/mL [GSD 2.77); *P* < 0.0001), and AY.12 (GM 12.52 U/mL [GSD 2.52] versus GM 8.13 U/mL [GSD, 2.83] *P* < 0.0001) Delta variant spike proteins (Fig. 2).

## DISCUSSION

We examined SARS-CoV-2 surrogates of virus neutralization and antibody concentrations 6 months after the initial SARS-CoV-2 vaccine, specifically examining the wild-type and multiple Delta variant strains, among 571 middle-aged adults who received two doses an mRNA vaccine. We found immune measures to be significantly higher in mRNA-1273-vaccined individuals, compared to the BNT162b2-vaccinated individuals with similar characteristics, regardless of the target virus strain tested.

Although efficacy of both the BNT162b2 (10) and mRNA-1273 (11) vaccines have been shown to remain > 95% against severe disease to at least 6 months, there are concerns that the effectiveness of the BNT162b2 vaccine may decrease with time against all-severity COVID-19 (12). Our immunogenicity data are consistent with a recent study demonstrating higher effectiveness of two doses of mRNA-1273, compared with two doses of BNT162b2, when Delta variant predominates (13). Collectively, these findings demonstrate higher magnitude 6-month humeral immunity with the mRNA-1273 vaccine, which may be explained by the higher relative mRNA dose (100 μg in the mRNA-1273 vaccine versus 30 μg in the BNT162b2 vaccine). Phase 1 and 2 trials of the mRNA-1273 vaccine reported that a mRNA dose of 100 μg demonstrated higher immunogenicity than a 25 μg dose, but not in comparison with a 50 μg dose (14).

Given ongoing global supply constraints, particularly of more efficacious mRNA vaccines, and that close to half of the global population has yet to be vaccinated, long-term immunogenicity should be considered when choosing vaccine types, especially for remote and/or marginalized populations where administration of third doses may prove challenging. These data may also have implications when contemplating timing of third vaccine doses for the general population, to prioritize individuals vaccinated initially with BNT162b2 (vs. mRNA-1273) vaccines.

Previous studies have compared immunogenicity elicited from the Delta versus wild-type strains, with many showing a decrease in neutralization (7, 15). While we did not directly compare immunogenicity between SARS-CoV-2 strain-based groups, ACE-2 inhibition from the wild-type and all Delta lineages were similar, as were comparisons between vaccine types within each group. These findings are congruent with previous work showing similar neutralization sensitivity of the Delta variant and its sublineages (8, 9). Overall, our results indicate that the mRNA-1273 vaccine similarly elicits a higher immune response for the wild-type and all tested Delta strains.

Our study is subject to several limitations. These data include individuals with 7-week (on average) intervals between first and second doses which may partially explain relatively high concentrations of 6-month antibodies and may differ if manufacturer recommended dosing schedules are used. Our study reports markers of humoral immune response, rather than clinical outcomes, which may not reflect all elements of protection and may not reflect clinical outcomes. We included middle-aged adult paramedics, whose immunity may differ from other groups. This was an observational study, which resulted in an uneven number of participants in the comparison groups. Conclusions are limited to association and confounders may have affected results. While the Delta variant accounted for over 99% of global COVID-19 at the time of this analysis (5), additional testing may be required to determine generalizability of these findings to new SARS-CoV-2 variants of concern such as Omicron.

In conclusion, we found that adults who received two doses of mRNA-1273 vaccines demonstrated higher wild-type and Delta variant-specific immunogenicity at 6 months, compared with those who received two doses of the BNT162b2 vaccine

## MATERIALS AND METHODS

### Parent study, study design, and setting.

The COVID-19 Occupational Risks, Seroprevalence and Immunity among Paramedics in Canada (CORSIP) study is a prospective observational cohort study of adult (≥ 19 years of age) paramedics working in the provinces of British Columbia, Alberta, Saskatchewan, Manitoba, and Ontario. The CORSIP study was approved by the University of British Columbia (H2O-03620) and University of Toronto (40435) research ethics boards and started enrolling participants in January 2021, with written consent. Participants completed sociodemographic and health questionnaires detailing vaccination status and history of nucleic acid amplification test (NAAT)-confirmed COVID-19 infections. Participants were requested to provide a blood sample at enrollment, and also 6 months after the first vaccine dose (if applicable). Data from this study can be obtained from the COVID-19 Immunity Task Force.

### Selection of participants.

For this analysis, we included participants who received two doses of the BNT162b2 vaccine or two doses of the mRNA-1273 vaccine, and provided a blood sample 170 to 190 days after the first vaccine dose. We excluded individuals with previous COVID-19, defined as a positive NAAT test or a reactive test on the Elecsys Anti-SARS-CoV-2 nucleocapsid (Roche, IND, USA; see supplemental materials) assay (which is indicative of prior SARS-CoV-2 infection rather than vaccination).

### Outcome measures.

We used several assays in this investigation to evaluate immunogenicity, reporting antibody and inhibition of angiotensin-converting enzyme 2 (ACE-2) binding concentrations to SARS-CoV-2 antigens. While SARS-CoV-2 live viral neutralization testing is typically viewed as the optimal surrogate measure of immune protection—as has been associated with COVID-19 severity (16–19)—neutralization testing procedures are labor-intensive, precluding large volume testing. Alternatively, ACE-2 inhibition and anti-spike antibody concentrations have been shown to strongly correlate with live virus neutralization assays (20–23).

The primary outcome in this study was spike IgG antibody concentrations from the prototypic wild-type SARS-CoV-2 strain, measured with the V-PLEX COVID-19 Panel 2 IgG assay (Meso Scale Discovery, MD, USA), reported in the standardized “binding antibody units” per milliliter (BAU/mL) (24). Secondary outcomes included: IgG antibody concentrations against the RBD from the wild-type SARS-CoV-2 strain, measured with the V-PLEX COVID-19 Panel 2 IgG assay (Meso Scale Discovery, MD, USA; reported in BAU/mL); wild-type spike total antibody concentrations measured with the Elecsys Anti-SARS-CoV-2 S total antibody assay (Roche, IND, USA; reported in BAU/mL); ACE-2 binding to the wild-type spike protein, and AY.1, AY.2, B.1.617.2/AY.3/AY.5/AY.6/AY.7/AY.14, B.1.617.2/AY.4, and AY.12 spike proteins from Delta variant subtypes (6), measured on the V-PLEX SARS-CoV-2 Panel 19 ACE2 Kit (Meso Scale Discovery, MD, USA; measured in units/mL [U/mL]). Delta proteins were chosen for inclusion in Panel 19 ACE2 Kit based on the predominant lineages, however do not represent all Delta lineage strains (6).

### Statistical analyses.

We performed analyses using GraphPad Prism Version 9.2.0 (GraphPad Software, San Diego, CA). For participant characteristics, we described categorical variables as counts (with percentages) and continuous variables as means (with standard deviation [SD]). We reported outcomes as geometric mean (GM) with geometric standard deviation (GSD), and compared using the Mann-Whitney U test. A 2-sided *P* value of <0.05 was classified as statistically significant.

## Supplementary Material

Reviewer comments

## References

[1] Polack FP, Thomas SJ, Kitchin N, Absalon J, Gurtman A, Lockhart S, Perez JL, Pérez Marc G, Moreira ED, Zerbini C, Bailey R, Swanson KA, Roychoudhury S, Koury K, Li P, Kalina WV, Cooper D, Frenck RW, Hammitt LL, Türeci Ö, Nell H, Schaefer A, Ünal S, Tresnan DB, Mather S, Dormitzer PR, Şahin U, Jansen KU, Gruber WC, C4591001 Clinical Trial Group. 2020. Safety and efficacy of the BNT162b2 mRNA COVID-19 vaccine. N Engl J Med 383:2603–2615. doi:10.1056/NEJMoa2034577. 33301246 PMC7745181

[2] Baden LR, El Sahly HM, Essink B, Kotloff K, Frey S, Novak R, Diemert D, Spector SA, Rouphael N, Creech CB, McGettigan J, Khetan S, Segall N, Solis J, Brosz A, Fierro C, Schwartz H, Neuzil K, Corey L, Gilbert P, Janes H, Follmann D, Marovich M, Mascola J, Polakowski L, Ledgerwood J, Graham BS, Bennett H, Pajon R, Knightly C, Leav B, Deng W, Zhou H, Han S, Ivarsson M, Miller J, Zaks T, Cove SG. 2021. Efficacy and safety of the mRNA-1273 SARS-CoV-2 vaccine. N Engl J Med 384:403–416. doi:10.1056/NEJMoa2035389. 33378609 PMC7787219

[3] World Health Organization. 2021. COVID-19 weekly epidemiological update. Edition 55. World Health Organization, Geneva, Switzerland. https://www.who.int/publications/m/item/weekly-epidemiological-update-on-covid-19---31-august-2021. Accessed August 31, 2021.

[4] Fisman DN, Tuite AR. 2021. Evaluation of the relative virulence of novel SARS-CoV-2 variants: a retrospective cohort study in Ontario, Canada. CMAJ 193:E1619–E1625. doi:10.1503/cmaj.211248. 34610919 PMC8562985

[5] World Health Organization. 2021. COVID-19 weekly epidemiological update. Edition 70 (December 14, 2022). World Health Organization, Geneva, Switzerland. https://www.who.int/publications/m/item/weekly-epidemiological-update-on-covid-19---11-january-2022. Accessed January 11, 2022.

[6] Pango lineages: Latest epidemiological lineages of SARS-CoV-2. 2021. cov-lineages.org. Accessed December 1, 2021.

[7] Planas D, Veyer D, Baidaliuk A, Staropoli I, Guivel-Benhassine F, Rajah MM, Planchais C, Porrot F, Robillard N, Puech J, Prot M, Gallais F, Gantner P, Velay A, Le Guen J, Kassis-Chikhani N, Edriss D, Belec L, Seve A, Courtellemont L, Péré H, Hocqueloux L, Fafi-Kremer S, Prazuck T, Mouquet H, Bruel T, Simon-Lorière E, Rey FA, Schwartz O. 2021. Reduced sensitivity of SARS-CoV-2 variant Delta to antibody neutralization. Nature 596:276–280. doi:10.1038/s41586-021-03777-9. 34237773

[8] Arora P, Kempf A, Nehlmeier I, Graichen L, Sidarovich A, Winkler MS, Schulz S, Jäck H-M, Stankov MV, Behrens GMN, Pöhlmann S, Hoffmann M. 2021. Delta variant (B.1.617.2) sublineages do not show increased neutralization resistance. Cell Mol Immunol 18:2557–2559. doi:10.1038/s41423-021-00772-y. 34635807 PMC8503871

[9] Lassaunière R, Polacek C, Fonager J, Bennedbæk M, Boding L, Rasmussen M, Fomsgaard A. 2021. Neutralisation of the SARS-CoV-2 Delta variant sub-lineages AY.4.2 and B.1.617.2 with the mutation E484K by Comirnaty (BNT162b2 mRNA) vaccine-elicited sera, Denmark, 1 to 26 November 2021. Eurosurveillance 26. doi:10.2807/1560-7917.ES.2021.26.49.2101059. PMC8662802 34886943

[10] Thomas SJ, Moreira ED, Kitchin N, Absalon J, Gurtman A, Lockhart S, Perez JL, Pérez Marc G, Polack FP, Zerbini C, Bailey R, Swanson KA, Xu X, Roychoudhury S, Koury K, Bouguermouh S, Kalina WV, Cooper D, Frenck RW, Hammitt LL, Türeci Ö, Nell H, Schaefer A, Ünal S, Yang Q, Liberator P, Tresnan DB, Mather S, Dormitzer PR, Şahin U, Gruber WC, Jansen KU, C4591001 Clinical Trial Group. 2021. Safety and efficacy of the BNT162b2 mRNA COVID-19 vaccine through 6 months. N Engl J Med 385:1761–1773. doi:10.1056/NEJMoa2110345. 34525277 PMC8461570

[11] El Sahly HM, Baden LR, Essink B, Doblecki-Lewis S, Martin JM, Anderson EJ, Campbell TB, Clark J, Jackson LA, Fichtenbaum CJ, Zervos M, Rankin B, Eder F, Feldman G, Kennelly C, Han-Conrad L, Levin M, Neuzil KM, Corey L, Gilbert P, Janes H, Follmann D, Marovich M, Polakowski L, Mascola JR, Ledgerwood JE, Graham BS, August A, Clouting H, Deng W, Han S, Leav B, Manzo D, Pajon R, Schödel F, Tomassini JE, Zhou H, Miller J. 2021. Efficacy of the mRNA-1273 SARS-CoV-2 vaccine at completion of blinded phase. N Engl J Med 385:1774–1785. doi:10.1056/NEJMoa2113017. 34551225 PMC8482810

[12] Chemaitelly H, Tang P, Hasan MR, AlMukdad S, Yassine HM, Benslimane FM, Al Khatib HA, Coyle P, Ayoub HH, Al Kanaani Z, Al Kuwari E, Jeremijenko A, Kaleeckal AH, Latif AN, Shaik RM, Abdul Rahim HF, Nasrallah GK, Al Kuwari MG, Al Romaihi HE, Butt AA, Al-Thani MH, Al Khal A, Bertollini R, Abu-Raddad LJ. 2021. Waning of BNT162b2 vaccine protection against SARS-CoV-2 infection in Qatar. N Engl J Med 385:e83. doi:10.1056/NEJMoa2114114. 34614327 PMC8522799

[13] Dickerman BA, Gerlovin H, Madenci AL, Kurgansky KE, Ferolito BR, Figueroa Muñiz MJ, Gagnon DR, Gaziano JM, Cho K, Casas JP, Hernán MA. 2022. Comparative effectiveness of BNT162b2 and mRNA-1273 vaccines in U.S. veterans. N Engl J Med 386:105–115. doi:10.1056/NEJMoa2115463. 34942066 PMC8693691

[14] Vaccines and related biological products advisory committee meeting. 2020. FDA briefing document, Moderna COVID-19 vaccine. https://www.fda.gov/media/144434/download. Accessed January 17, 2022. 10.1016/j.vaccine.2019.10.048 31706809

[15] Davis C, Logan N, Tyson G, Orton R, Harvey WT, Perkins JS, Mollett G, Blacow RM, Peacock TP, Barclay WS, Cherepanov P, Palmarini M, Murcia PR, Patel AH, Robertson DL, Haughney J, Thomson EC, Willett BJ, COVID-19 DeplOyed VaccinE (DOVE) Cohort Study investigators. 2021. Reduced neutralisation of the Delta (B.1.617.2) SARS-CoV-2 variant of concern following vaccination. PLoS Pathog 17:e1010022. doi:10.1371/journal.ppat.1010022. 34855916 PMC8639073

[16] McMahan K, Yu J, Mercado NB, Loos C, Tostanoski LH, Chandrashekar A, Liu J, Peter L, Atyeo C, Zhu A, Bondzie EA, Dagotto G, Gebre MS, Jacob-Dolan C, Li Z, Nampanya F, Patel S, Pessaint L, Van Ry A, Blade K, Yalley-Ogunro J, Cabus M, Brown R, Cook A, Teow E, Andersen H, Lewis MG, Lauffenburger DA, Alter G, Barouch DH. 2021. Correlates of protection against SARS-CoV-2 in rhesus macaques. Nature 590:630–634. doi:10.1038/s41586-020-03041-6. 33276369 PMC7906955

[17] Dispinseri S, Secchi M, Pirillo MF, Tolazzi M, Borghi M, Brigatti C, De Angelis ML, Baratella M, Bazzigaluppi E, Venturi G, Sironi F, Canitano A, Marzinotto I, Tresoldi C, Ciceri F, Piemonti L, Negri D, Cara A, Lampasona V, Scarlatti G. 2021. Neutralizing antibody responses to SARS-CoV-2 in symptomatic COVID-19 is persistent and critical for survival. Nat Commun 12:2670. doi:10.1038/s41467-021-22958-8. 33976165 PMC8113594

[18] Feng S, Phillips DJ, White T, Sayal H, Aley PK, Bibi S, Dold C, Fuskova M, Gilbert SC, Hirsch I, Humphries HE, Jepson B, Kelly EJ, Plested E, Shoemaker K, Thomas KM, Vekemans J, Villafana TL, Lambe T, Pollard AJ, Voysey M, Oxford COVID Vaccine Trial Group. 2021. Correlates of protection against symptomatic and asymptomatic SARS-CoV-2 infection. Nat Med 27:2032–2040. doi:10.1038/s41591-021-01540-1. 34588689 PMC8604724

[19] Bergwerk M, Gonen T, Lustig Y, Amit S, Lipsitch M, Cohen C, Mandelboim M, Levin EG, Rubin C, Indenbaum V, Tal I, Zavitan M, Zuckerman N, Bar-Chaim A, Kreiss Y, Regev-Yochay G. 2021. Covid-19 breakthrough infections in vaccinated health care workers. N Engl J Med 385:1474–1484. doi:10.1056/NEJMoa2109072. 34320281 PMC8362591

[20] Tan CW, Chia WN, Qin X, Liu P, Chen MI-C, Tiu C, Hu Z, Chen VC-W, Young BE, Sia WR, Tan Y-J, Foo R, Yi Y, Lye DC, Anderson DE, Wang L-F. 2020. A SARS-CoV-2 surrogate virus neutralization test based on antibody-mediated blockage of ACE2-spike protein-protein interaction. Nat Biotechnol 38:1073–1078. doi:10.1038/s41587-020-0631-z. 32704169

[21] Abe KT, Li Z, Samson R, Samavarchi-Tehrani P, Valcourt EJ, Wood H, Budylowski P, Dupuis AP, Girardin RC, Rathod B, Wang JH, Barrios-Rodiles M, Colwill K, McGeer AJ, Mubareka S, Gommerman JL, Durocher Y, Ostrowski M, McDonough KA, Drebot MA, Drews SJ, Rini JM, Gingras A-C. 2020. A simple protein-based surrogate neutralization assay for SARS-CoV-2. JCI Insight 5. doi:10.1172/jci.insight.142362. PMC7566699 32870820

[22] Dolscheid‐Pommerich R, Bartok E, Renn M, Kümmerer BM, Schulte B, Schmithausen RM, Stoffel‐Wagner B, Streeck H, Saschenbrecker S, Steinhagen K, Hartmann G. 2022. Correlation between a quantitative anti‐SARS‐CoV‐2 IgG ELISA and neutralization activity. J Med Virol 94:388–392. doi:10.1002/jmv.27287. 34415572 PMC8426838

[23] Tea F, Ospina Stella A, Aggarwal A, Ross Darley D, Pilli D, Vitale D, Merheb V, Lee FXZ, Cunningham P, Walker GJ, Fichter C, Brown DA, Rawlinson WD, Isaacs SR, Mathivanan V, Hoffmann M, Pöhlman S, Mazigi O, Christ D, Dwyer DE, Rockett RJ, Sintchenko V, Hoad VC, Irving DO, Dore GJ, Gosbell IB, Kelleher AD, Matthews GV, Brilot F, Turville SG. 2021. SARS-CoV-2 neutralizing antibodies: longevity, breadth, and evasion by emerging viral variants. PLoS Med 18:e1003656. doi:10.1371/journal.pmed.1003656. 34228725 PMC8291755

[24] Kristiansen PA, Page M, Bernasconi V, Mattiuzzo G, Dull P, Makar K, Plotkin S, Knezevic I. 2021. WHO international standard for anti-SARS-CoV-2 immunoglobulin. Lancet 397:1347–1348. doi:10.1016/S0140-6736(21)00527-4. 33770519 PMC7987302

