# Supplementary material for: Comparative 6-Month Wild-Type and Delta-Variant Antibody Levels and Surrogate Neutralization for Adults Vaccinated with BNT162b2 versus mRNA-1273
Source: Microbiol Spectr. 2022 Mar 7;10(2):e02702-21. doi: 10.1128/spectrum.02702-21 (PMC9045183; doi:10.1128/spectrum.02702-21)
Supplement: SUPPLEMENTAL FILE 1 — Supplemental material. Download SPECTRUM02702-21_Supp_1_seq3.pdf, PDF file, 0.1 MB [file spectrum02702-21_supp_1_seq3.pdf]

SUPPLEMENTAL MATERIALS

V-PLEX ACE-2 Inhibition Testing Procedures

Inhibition of human ACE-2 receptor binding onto viral spike was measured using the V-PLEX SARS-COV2 Panel 19 ACE-2 kit (Meso Scale Discovery [MSD], MD, USA; reported as Units per mL), for the Wuhan virus strain and the following lineages (and amino acid modifications):

- AY.1 (T19R, T95I, G142D, E156G, Δ157/158, W258L, K417N, L452R, T478K, D614G, P681R, D950N)
- AY.2 (T19R, V70F, G142D, E156G, Δ157/158, A222V, K417N, L452R, T478K, D614G, P681R, D950N)
- B.1.617.2/AY.3/AY.5/AY.6/AY.7/AY.14 (T19R, G142D, Δ156/157, R158G, L452R, T478K, D614G, P681R, D950N)
- B.1.617.2/AY.4 (T19R, T95I, G142D, Δ156/157, R158G, L452R, T478K, D614G, P681R, D950N)
- AY.12 (T19R, Δ156/157, R158G, L452R, T478K, D614G, P681R, D950N)

The MSD assay was performed as per the manufacturer's instructions, at the BC Children's Hospital Research Institute (Golding, Prusinkiewicz, and Lavoie). Samples were tested at a 1:10,000 dilution, with the diluent provided by the manufacturer (Diluent 100).

V-PLEX Spike and Receptor-Binding Domain Antibody Testing Procedures

Anti-S1 spike and Receptor-Binding Domain (RBD) IgG antibody concentrations were measured using the V-PLEX COVID-19 Coronavirus Panel 2 IgG assay (MSD, MD, USA). The MSD assay was performed as per the manufacturer's instructions, at the BC Centre for Disease Control (Marquez and Jassem). Samples were tested at a 1:5000 dilution, with the diluent provided by the manufacturer (Diluent 100). Heat inactivation was not performed. The assay includes a standard curve based on a reference standard that contains a pre-determined concentration of each antigen. It also includes three serological controls which contain a known concentration of IgG antibodies against the antigens of the assay. We ran the standard curve and serological controls on every plate to assess for quality control. Results were initially reported as arbitrary units per mL, and then converted to the internationally standardized "binding antibody units" (BAU)[1] with a conversion factor of 0.00901 for spike and 0.0272 for RBD.[2]

Elecsys Spike and Nucleocapsid Antibody Testing Procedures

Anti-S1 spike was measured with the quantitative Elecsys Anti-SARS-Cov-2 S total antibody assay (Roche, IND, USA).[3] The assay was performed as per manufacturer's instructions, with lot-to-lot standardization and assay calibration as per Roche standard operating procedures and manufacturer's instructions. Samples with an anti-S concentration above the measuring range (250 U/ml) were diluted by the Roche analyzer with Universal Diluent at 1:400. After dilution by the analyzer, the software automatically utilized the dilution value when calculating the sample concentration. Heat inactivation was not performed as was not part of the manufacturer's standard protocol. For quality control, PreciControl Anti-SARS-CoV-2 was used as per manufacturer's instructions. These were run at least daily prior to any testing (e.g., every 24 hours when test is in use). Cut-offs were determined automatically by the analyzer software

based on calibrated master curves. For calibration, the method is standardized against an internal Roche standard for anti-SARS-CoV-2 provided with the assay (N and S). A pre-defined master curve is adapted to the analyzer using the kit calibration reagents. Calibration is performed as per manufacturer's instructions. Results were initially reported as units per mL, and then converted to standardized BAU with a conversion factor of 1.288.[4]

All serum samples were also similarly tested with the Elecsys Anti-SARS-CoV-2 nucleocapsid (Roche, IND, USA) assay[5], an immunoassay for the in-vitro qualitative detection of nucleocapsid antibodies (including IgG) to SARS-CoV-2, in order to determine eligibility. Both Elecsys tests were performed at the Canadian Blood Services national COVID-19 research laboratory (Drews and O'Brien).

References

1. Kristiansen PA, Page M, Bernasconi V, Mattiuzzo G, Dull P, Makar K, Plotkin S, Knezevic I. WHO International Standard for anti-SARS-CoV-2 immunoglobulin. *Lancet*. 2021;397(10282):1347-1348. doi:10.1016/S0140-6736(21)00527-4.
2. Meso Scale Discovery. MSD Reference Standard. https://www.mesoscale.com/~/media/files/product inserts/reference standard 1 insert.pdf. Published 2021. Accessed January 18, 2022.
3. Cobas. Elecsys Anti-SARS-CoV-2 S V 1.0. https://www.rochecanada.com/content/dam/rochexx/roche-ca/products/docs/package_inserts/Anti-SARS-CoV-2-S-09289267190-EN-Can.pdf. Published 2021. Accessed August 10, 2021.
4. Infantino M, Pieri M, Nuccetelli M, Grossi V, Lari B, Tomassetti F, Calugi G, Pancani S, Benucci M, Casprini P, Manfredi M, Bernardini S. The WHO International Standard for COVID-19 serological tests: towards harmonization of anti-spike assays. *Int Immunopharmacol*. 2021;100:108095. doi:10.1016/j.intimp.2021.108095.
5. Ainsworth M, Andersson M, Auckland K, Baillie JK, Barnes E, Beer S, Beveridge A, Bibi S, Young RK, et al. Performance characteristics of five immunoassays for SARS-CoV-2: a head-to-head benchmark comparison. *Lancet Infect Dis*. 2020;20(12):1390-1400. doi:10.1016/S1473-3099(20)30634-4.
